# Supplementary material for: Swept coded aperture real-time femtophotography
Source: Nat Commun. 2024 Feb 21;15:1589. doi: 10.1038/s41467-024-45820-z (PMC10882056; doi:10.1038/s41467-024-45820-z)
Supplement: Supplementary file 1 — Supplementary Information [file 41467_2024_45820_MOESM1_ESM.pdf]

SUPPLEMENTARY MATERIALS

Swept coded aperture real-time femtophotography

Jingdan Liu[1,†,‡], Miguel Marquez[1,†], Yingming Lai[1], Heide Ibrahim[1], Katherine Légaré[1], Philippe Lassonde [1], Xianglei Liu[1], Michel Hehn[2], Stéphane Mangin[2], Grégory Malinowski[2], Zhengyan Li[3], François Légaré[1], Jinyang Liang[1,*]

[1]Centre Énergie Matériaux Télécommunications, Institut National de la Recherche Scientifique, Université du Québec, 1650 boulevard Lionel-Boulet, Varennes, Québec J3X1P7, CANADA

[2]Institut Jean Lamour, Université de Lorraine, Parc de Saurupt CS 50840, Nancy 54011, FRANCE

[3]School of Optical and Electronic Information, Huazhong University of Science and Technology, 1037 Luoyu Road, Wuhan, Hubei 430074, CHINA

[†]These authors contributed equally to this work

[‡]Present address: Shanghai Institute of Optics and Fine Mechanics, Chinese Academy of Sciences, Shanghai 201800, CHINA.

*Corresponding author: jinyang.liang@inrs.ca

Supplementary Note 1: Derivation of SCARF's data acquisition

In data acquisition, swept coded aperture real-time femtophotography (SCARF) compressively records an (x, y, t) dynamic scene into a two-dimensional (2D) snapshot (Fig. 1). For simplicity of notation, we here make the following assumptions. First, the imaging system has a unit magnification. Second, the dynamic scene can be perfectly imaged throughout the system. Third, the chirped pulse has a perfect linear relationship between wavelength and time. Fourth, the binned pixel of the deployed camera and the encoding mask have the same pixel size, denoted by d.

In the following, we first derive the expression of SCARF's data acquisition in the continuous model and then discretize it for the image reconstruction algorithm. For an ultrashort laser pulse with a temporal linear chirp, the relationship between time t and wavelength λ can be expressed as [1]

$$\lambda = \lambda_0 + (t - t_0)\frac{\lambda_0^2}{2\pi c\varphi''}, \tag{S1}$$

where λ_0 is the central wavelength of the chirped laser pulse, and t_0 is the time point that corresponds to the central wavelength. For a narrow spectral band, we only consider up to second-order dispersion in the optical element, which is represented as φ''. We further define $\beta = 2\pi c\varphi''/\lambda_0^2$ as linear time-spectrum mapping parameter, where c is the vacuum speed of light. Equation (S1) governs the linear mapping between time and wavelengths, which is denoted by the operator $\mathbf{M}$. Hence, if a dynamic event, whose transmittance to a linear chirped probe pulse is $a(x, y, t)$, the spectral density of the transmitted light is expressed by

$$I_\mathrm{M}(x, y, \lambda) = \mathbf{M}\{a(x, y, t)\}\,. \tag{S2}$$

Next, $I_\mathrm{M}(x, y, \lambda)$ incidents into the modified pulse shaper (Fig. 1a). The first dispersive $4f$ imaging system, consisting of lenses L1 and L2 and the grating G1, images the transient scene onto a pseudo-random binary transmissive mask with a spectral shearing operation, denoted by **S**. The spectral density just before this encoding mask is expressed by

$$\begin{aligned} I_\mathrm{S}(x, y, \lambda) &= \mathbf{S}\{I_\mathrm{M}(x, y, \lambda)\} \\ &= \iint dx'dy'\delta(x' - [x + \alpha f_2(\lambda - \lambda_0)])\delta(y' - y)I_\mathrm{M}(x', y', \lambda) \\ &= I_\mathrm{M}(x + \alpha f_2(\lambda - \lambda_0), y, \lambda)\,. \end{aligned} \tag{S3}$$

Here, $\delta(\cdot)$ stands for the Dirac delta function, α is the angular dispersion of the grating G1, and f_2 is the focal length of Lens 2.

Then, the mask spatially encodes $I_\mathrm{S}(x, y, \lambda)$. Because random binary matrices are largely incoherent with any fixed basis [2], they are used as a universal coded aperture in SCARF. The spectral density immediately after this coded aperture is given by

$$\begin{aligned} I_\mathrm{C}(x, y, \lambda) &= \mathbf{C}\{I_\mathrm{S}(x, y, \lambda)\} \\ &= c(x, y) \cdot I_\mathrm{M}(x + \alpha f_2(\lambda - \lambda_0), y, \lambda)\,, \end{aligned} \tag{S4}$$

where **C** stands for the spatial encoding operator. $c(x, y)$ represents the spatial transmittance of the pseudo-random binary pattern. Equations (S3)–(S4) reveal that the different wavelength components of the incident light are smeared to different spatial positions and thus are encoded by different portions of the mask.

Behind the coded aperture, $I_{\mathrm{C}}(x,y,\lambda)$ passes through another dispersive $4f$ imaging system (consisting of lenses L3 and L4 and the grating G2) that is symmetric to the first one. This process results in another spectral shearing operation (denoted by $\mathbf{S}'$), whose shearing direction is opposite to that of $\mathbf{S}$. The spectral density of light entering the sensor is expressed by

$$\begin{aligned} I_{\mathrm{S}'}(x,y,\lambda) &= \mathbf{S}'\{I_{\mathrm{C}}(x,y,\lambda)\} \\ &= \iint dx'dy'\delta(x'-[x-\alpha f_4(\lambda-\lambda_0)])\delta(y'-y)I_{\mathrm{C}}(x',y',\lambda) \\ &= c(x-\alpha f_4(\lambda-\lambda_0),y,\lambda)I_{\mathrm{M}}(x,y,\lambda)\,. \end{aligned} \tag{S5}$$

Here, f_4 is the focal length of Lens 4, and $f_4 = f_2$. The first term in Eq. (S5), $c(x-\alpha f_4(\lambda-\lambda_0),y,\lambda)$, reveals that the 2D coded aperture sweeps through the three-dimensional (3D) datacube for spatial encoding. The second term, $I_{\mathrm{M}}(x,y,\lambda)$, shows that no shearing effect exists on the transmitted light.

On the sensor, $I_{\mathrm{S}'}(x,y,\lambda)$ is recorded via spatiotemporal integration (i.e., spatially integrated over each sensor pixel and temporally integrated over the duration of the transient event) that is denoted by $\mathbf{T}$. The optical energy measured at pixel $[m,n]$ is given by

$$\begin{aligned} E[m,n] &= \mathbf{T}\,\{I_{S'}(x,y,\lambda)\} \\ &= \int dx \int dy \left\{\left[\iint d\lambda\, I_{\mathrm{S}'}(x,y,\lambda)\right] \mathrm{rect}\left(\frac{x}{d}-\left(m+\frac{1}{2}\right),\frac{y}{d}-\left(n+\frac{1}{2}\right)\right)\right\}. \end{aligned} \tag{S6}$$

Equations (S2)–(S6) describe a continuous-to-discrete forward model for the SCARF system. However, to use this forward model numerically, a discrete-to-discrete model needs to be derived. We choose a voxel of $(d,d,\Delta\lambda_{\mathrm{FI}},\tau_{\mathrm{FI}})$ in the x-y-λ-t space. Here, “FI” stands for “frame interval”. $\Delta\lambda_{\mathrm{FI}}$ is defined by

$$\Delta\lambda_{\mathrm{FI}} = \frac{d}{f_2\alpha}. \tag{S7}$$

Correspondingly, τ_{FI} is defined by

$$\tau_{\mathrm{FI}} = \beta\,\Delta\lambda_{\mathrm{FI}}\,. \tag{S8}$$

In this way, the discrete form of the dynamic scene can be expressed as

$$a[m,n,k] = \int dt \int dx \int dy\ a(x,y,t)\ \mathrm{rect}\left(\frac{x}{d} - \left(m + \frac{1}{2}\right), \frac{y}{d} - \left(n + \frac{1}{2}\right), \frac{t}{\tau_{\mathrm{FI}}} - \left(k + \frac{1}{2}\right)\right). \qquad \text{(S9)}$$

The encoding mask, which can be discretized similarly to Eq. (S9), is denoted as $c[m,n]$. Therefore, Eq. (S6), representing the imaging model of the SCARF system, can be rewritten in a discrete form as

$$E[m,n] = \frac{d^3\beta}{\alpha f_2} \sum_k I_{\mathrm{C}}[m-k,n,k]\,. \qquad \text{(S10)}$$

Equation (S10) is implemented in SCARF's image reconstruction.

The derivation of SCARF's data acquisition can be further elaborated with the following notes and explanations. Equations (S7)–(S10) show that $x, y,$ and t information of the dynamic scene is discretized based on the binned pixel size of the deployed camera, i.e., d. This criterion determines the dimensions of the datacube of the dynamic scene, which are denoted by $N_x \times N_y \times N_t$. After the spectral shearing operation (i.e., $\mathbf{S}$), the size of the datacube is changed to $(N_x + N_t - 1) \times N_y \times N_t$, which shows that the adjacent frames are shifted by one binned camera pixel in the x direction [Eq. (S4)]. In the subsequent spatial encoding operation, the random mask has a size of $(N_x + N_t - 1) \times N_y$. Thus, each frame is encoded by a different part of the mask [Eq. (S5)]. Finally, the dispersion completely cancels after the second spectral shearing operation (i.e., $\mathbf{S}'$), leaving the datacube with a size of $N_x \times N_y \times N_t$. After the spatiotemporal integration, the captured snapshot has a size of $N_x \times N_y$. As a result, the maximum frame size in the reconstructed video is limited by the deployed camera, i.e., $N_x \leq N_{\mathrm{col}}$ and $N_y \leq N_{\mathrm{row}}$, where N_{col} and N_{row} are the number of columns and rows of the deployed camera.

SCARF's compressed ultrafast imaging capability is supported by the pseudo-random mask used in the spatial encoding operation and the ensuing spectral shearing operation (i.e., $\mathbf{S}'$). In a binary pseudo-random mask, the cross-correlation of any two different columns is 0.5, which means that they are linearly independent. Because only one spectral shearing operation is conducted after the spatial encoding operation, these different parts in the pseudo-random encoding mask, imparted with each frame, linearly shift and overlap. As a result, each column of the captured snapshot is the integration of the corresponding columns of each frame multiplied by different columns of the random encoding mask. Therefore, this column in the snapshot can be

regarded as the summation of N_t linearly independent measurements. This framework thus provides a theoretical foundation for the ensuing image reconstruction to extract spatiotemporal information from the snapshot.

Equation (S8) defines the time interval between the two adjacent frames. In this way, SCARF's imaging speed can be defined by $r = 1/\tau_{\mathrm{FI}} = f_2\alpha/d\beta$, which is related to the dispersion capability of the diffraction grating, the duration of the chirp pulse, the camera sensor's pixel size, and the focal length of Lens 2. An additional explanation of its link to the sweeping speed of the coded aperture is provided in Supplementary Note 4.

Supplementary Note 2: Details of SCARF's reconstruction algorithm

SCARF uses the plug-and-play (PnP) alternating direction method of multipliers (ADMM) framework for image reconstruction. To retrieve the transmittance of the dynamic scene $a(x, y, t)$, the inverse problem [i.e., Eq. (2) in the Main Text] is first written as

$$\begin{gathered}\hat{\mathbf{a}} = \underset{\mathbf{a},\mathbf{v},\mathbf{u},\mathbf{w}\in\mathcal{A}}{\operatorname{argmin}} \frac{1}{2}\|\mathbf{Tv} - \mathbf{E}\|_2^2 + \mathrm{R}(\mathbf{u}) + \mathrm{I}_+(\mathbf{w}) \\ \mathrm{s.t.}\ \ \mathbf{v} = \mathbf{O}'\mathbf{a}, \mathbf{u} = \mathbf{a}, \mathbf{w} = \mathbf{a},\end{gathered} \tag{S11}$$

where $\mathbf{v}$, $\mathbf{u}$, and $\mathbf{w}$ are primal variables and the operator $\mathbf{O}' = \mathbf{S}'\mathbf{CSM}$. $\mathbf{a}$ is the discrete version of $a(x, y, t)$. In addition, $\mathcal{A}$ represents a set of solutions that satisfy the spatial constraint generated by binarizing the snapshot image $\mathbf{E}$ with an appropriate intensity threshold determined by Otsu's method [3,4].

Then, Eq. (S11) is written in the augmented Lagrangian arguments

$$\begin{aligned}\hat{\mathbf{a}} = \underset{\mathbf{a},\mathbf{v},\mathbf{u},\mathbf{w}\in\mathcal{A}}{\operatorname{argmin}} & \frac{1}{2}\|\mathbf{Tv} - \mathbf{E}\|_2^2 + \mathrm{R}(\mathbf{u}) + \mathrm{I}_+(\mathbf{w}) \\ & + \frac{\mu_1}{2}\left\|\mathbf{O}'\mathbf{a} - \mathbf{v} + \frac{\boldsymbol{\gamma}_1}{\mu_1}\right\|_2^2 + \frac{\mu_2}{2}\left\|\mathbf{a} - \mathbf{u} + \frac{\boldsymbol{\gamma}_2}{\mu_2}\right\|_2^2 + \frac{\mu_3}{2}\left\|\mathbf{a} - \mathbf{w} + \frac{\boldsymbol{\gamma}_3}{\mu_3}\right\|_2^2 .\end{aligned} \tag{S12}$$

Here, $\boldsymbol{\gamma}_1$, $\boldsymbol{\gamma}_2$, and $\boldsymbol{\gamma}_3$ are dual variables. μ_1, μ_2, and μ_3 are penalty parameters [5]. The ramp function is used in the non-negative indicator function $\mathrm{I}_+(\cdot)$. The block-matching and 3D filtering (BM3D) [6] is used as the PnP denoiser in the implicit regularizer $\mathrm{R}(\cdot)$. BM3D is an advanced denoising method based on effective filtering in a 3D transform domain by combining the sliding-window transform process with block-matching [7]. In the sliding process, blocks with similar spatial features and intensity levels are selected using the block-matching concept [8]. These matched blocks are stacked to form a 3D array, and the data in the array exhibit high correlation. Then, a 3D de-

correlating unitary transformation is applied to exploiting this correlation and effectively attenuating the noise by reducing the transform coefficients. Finally, using an inverse 3D transformation, all matched blocks are estimated. This procedure is repeated for each sliding window, and the final estimate is computed as a weighted average of all of those overlapping estimates.

Next, the algorithm iteratively updates primal variables, the estimated solution $\mathbf{a}^{j+1}$ (j denotes the iteration index), and penalty parameters [9]. The algorithm stops once the following two conditions about the estimated results and penalty parameters are met:

$$\eta = \frac{\left\|\mathbf{a}^{j+1} - \mathbf{a}^{j}\right\|_2}{\left\|\mathbf{a}^{j}\right\|_2} < \rho \text{ and} \tag{S13.1}$$

$$\mu_i^{j+1} = \mu_i^{j} \ (i = 1, 2, 3)\ . \tag{S13.2}$$

Here, ρ ($0 < \rho \leq 10^{-3}$) is the pre-set tolerance value.

Supplementary Note 3: Details on the calibration of SCARF's spatial encoding operation

Using the operations of time-spectrum mapping, spectral shearing, and spatial encoding, SCARF tags each frame of the dynamic scene with a unique "barcode," which provides the prior information that allows the spatiospectral mixing of adjacent frames to be recovered by the PnP-ADMM algorithm. Due to various imperfections induced in fabrication, the actual encoding mask is different from the binary mask in design. Therefore, calibration of spatial encoding operation is necessary to obtain accurate information for the operator $\mathbf{C}$ in the image reconstruction.

This calibration was conducted using the following method. As shown in Supplementary Fig. 1a, we built an additional beamline with a continuous-wave (CW) diode laser (MDL-III-793nm-2W, CNI), whose output wavelength was tunable from 787.96 nm to 791.72 nm. The beam went through a spatial filtering stage to generate a Gaussian beam, whose size was similar to that of the ultrafast laser pulse. Then, we coupled this illumination into the SCARF system and aligned the paths of the CW laser beam to be collinear with that of the chirped ultrashort pulse.

We acquired the images of the coded aperture at five wavelengths of the CW laser beam, all of which were within the spectrum range of the chirped ultrashort probe pulse. After binarization, these images were used as $c[m, n]$ for image reconstruction. As shown in Supplementary Fig. 1b, these mask images have a high resemblance, suggesting a negligible

distortion. Besides, we calculated the shift in the spectral shearing direction versus calibration wavelengths using a cross-correlation-based image registration algorithm [10]. As shown in Supplementary Fig. 1c, this result proves the linear temporal shearing of the SCARF system.

Supplementary Note 4: Comparison of SCARF with representative techniques in single-shot compressed temporal imaging

The comparison of SCARF with four representative techniques in single-shot compressed temporal imaging is summarized in Supplementary Table 2. In particular, both SCARF and coded-aperture compressive temporal imaging (CACTI) [11-17] use full-sequence pixel-wise encoding to record temporal information. Their acquired snapshots have the same compression ratio, and their reconstructed movies have similar sequence depths. However, CACTI uses a moving translation stage [13,14] or a spatial light modulator [11,12] to generate the sweeping coded aperture, so that to our knowledge, its highest imaging speed ever demonstrated is limited to ~5 kfps [13]. In contrast, leveraging the difference in the number of shearing operations that are applied to the scene and the mask, SCARF allows the sweeping of a static coded aperture at an ultrafast speed. In particular, based on Eq. (S8), this sweeping speed can be defined by $v_{\mathrm{s}} = \frac{f_2 \alpha}{\beta}$, and SCARF's frame rate can thus be determined by $r = v_{\mathrm{s}}/d$. Given the specifications listed in Methods and Supplementary Table 1, the highest sweeping speed is calculated to be $v_{\mathrm{s}}=1.7\times10^9$ m/s, which leads to SCARF's highest imaging speed of r=156.3 Tfps. This approach enables SCARF to increase the imaging speed for more than 10 orders of magnitude compared to CACTI. Finally, we use a metric termed data acquisition capability, defined as $F = rN_t$, to synthetically consider the imaging speed and the sequence depth. A larger value of F indicates the system can capture a longer sequence depth at a higher imaging speed. Using this metric, compared to CACTI, SCARF improves the data acquisition capability by 2.3×10^9 times (for imaging ultrafast dynamics of a static object) and 2.1×10^{10} times (for imaging an ultrafast moving object).

The multiple-aperture compressed sensing (MA-CS) CMOS sensor [18] uses a 5 × 3 lens array to optically generate a total of 15 replicated images of the dynamic scene. Each replicated image is formed onto a CMOS sensor (64 × 108 pixels). Each CMOS sensor uses a unique binary random code sequence as a flutter shutter for temporal modulation. A compressed sensing-based algorithm is used to produce a movie with an imaging speed of r =200 million fps and a sequence

depth of N_t =32, which leads to F =6.4×10^{-3} frames · Tfps. Thus, compared to SCARF, the MA-CS CMOS technique has considerably lower specifications in sequence depth, compression ratio, frame size, and data acquisition capability.

Compressed ultrafast spectral-temporal (CUST) photography [19] and compressed ultrafast spectral photography (CUSP) [20] are developed based on the imaging model of compressed ultrafast photography (CUP) [21]. Similar to SCARF, both CUST photography and CUSP use chirped pulse illumination and a static coded aperture for spatial encoding to achieve imaging speeds at the Tfps level. However, in their models of data acquisition, only a single spectral shearing operation is applied to both the dynamic scene and the coded aperture. Consequently, pixel-wise encoding with the same depth cannot be realized across the field of view. As a result, their sensing matrices (and the one for CUP in general) do not possess a full-row rank property, thus resulting in a non-trivial null space for $\mathbf{OO}^{\mathrm{T}}$, which reduces the reconstruction speed and quality. Meanwhile, shearing the dynamic scene induces anisotropy in spatial resolutions and loses the prior knowledge to delineate the region of the dynamic scene [22]. Finally, this data acquisition scheme couples the frame size with the sequence depth, which caps the system's information acquisition capacity and reduces the compression ratio [22]. In contrast, SCARF applies the spatial encoding operation between two spectral shearing operations in opposite directions. SCARF's image acquisition paradigm successfully breaks all aforementioned limitations. The knowledge of the region of the dynamic scene and the preservation of the isotropy in spatial resolutions enhance the reconstructed image quality. Practically, SCARF exceeds the imaging speed of CUST by >40 times. Compared to CUSP, the all-optical SCARF does not need a streak camera, which avoids the degraded image quality in the captured snapshot due to various drawbacks and limitations imposed by the imaging using photoelectrons. It is worth noting that CUSP uses a pulse train that contains seven chirped sub-pulses for active illumination. Each chirped sub-pulse provides 140 frames and hence 980 frames for the pulse train. In Supplementary Table 2, both numbers are listed for sequence depth and are used for the calculation of data acquisition capability.

To better comprehend the influence of these sensing-matrix characteristics and further demonstrate the superior performance of SCARF to CUP, we compared the reconstructed image qualities using two simulated dynamic events. First, we simulated a static spoke pattern whose intensity changed as a Gaussian function (Supplementary Fig. 2a). The event datacube had dimensions of 512×512×21 pixels. The simulated snapshots using the forward models of CUP and

SCARF are shown in Supplementary Figs. 2b-c, respectively. Also shown as insets in Supplementary Figs. 2a–c are the zoom-in views of the center of these images. The reconstructed images are compared in Supplementary Figs. 2d–e, which show the 11th frames of the reconstructions of CUP and SCARF. To further evaluate the performance, we analyzed their noise-limited bandwidth at the 3σ threshold above the average background, where σ is the noise defined by the standard deviation of the background. These results were compared with the ground truth, as shown in Supplementary Figs. 2f–h. The results reveal that SCARF's spatial resolution decreases by 12.3% in both the x and the y directions. In comparison, CUP's spatial resolution decreases by 13.1% in the x direction and 48.6% in the y direction. Therefore, compared to CUP, SCARF has a higher spatial resolution and avoids resolution anisotropy. Finally, to quantitatively assess the quality of reconstructed videos, we analyzed the structural similarity (SSIM) index and peak signal-to-noise ratio (PSNR) frame by frame (Supplementary Figs. 2i–j). The SCARF reconstruction shows a better performance in each frame in both metrics. Compared to CUP, SCARF improves the SSIM index by 2.2% on average and enhances the PSNR by 2.0 dB on average.

To further investigate the superiority of SCARF against CUP, we simulated dynamic absorption in an elliptical ring pattern. Six representative images of this dynamic scene (256×300×22 pixels in size) are shown in the first row of Supplementary Fig. 3a. Notably, the ring pattern occupies the entire (x, y) frame. The corresponding frames of the datacubes retrieved by CUP and SCARF are shown in the second and the third rows in Supplementary Fig. 3a. The results, echoing Supplementary Fig. 2, show SCARF's capability to preserve all spatial features along the temporal dimension. In contrast, due to the mixture of spatial and temporal information in the shearing direction, CUP inevitably fails to image the entire spatial structure. In contrast, since its sensing paradigm can effectively mitigate the issues generated by single temporal shearing, SCARF produces superior image quality in all reconstructed frames with respect to CUP reconstructions (Supplementary Fig. 3b), which is quantitatively shown as a higher SSIM index of $14.0 \pm 1.2\%$.

Finally, to elucidate the advantages of the SCARF's sensing matrix conditionality, we analyzed convergence in the reconstruction algorithm using the logarithmic residual norm. As shown in Supplementary Fig. 3c, SCARF has an error of almost five orders of magnitude lower than CUP after 1000 iterations in the PnP-ADMM algorithm. SCARF's reconstruction and

convergence improvements (compared to CUP) are associated with the sensing matrix having a full-row rank property (see Supplementary Fig. 3d), thus resulting in a trivial null space for $\mathbf{O}\mathbf{O}^{\mathrm{T}}$. In the context of convex optimization, matrices characterized by a trivial null space facilitate the convergence of optimization algorithms. By minimizing redundancy and ambiguity in measurements, these matrices promote the efficient and reliable convergence of iterative algorithms, ultimately leading to quicker and more precise solutions. Finally, the SCARF's sensing matrix conditionality, thus the reconstruction performance, is analyzed by calculating the ℓ_2-norm error of the reconstruction obtained by the ADMM's closed-form solution [23] for the first iteration (i.e., $\tilde{\sigma}^{-1}[\boldsymbol{\Xi} - \mathbf{O}^{T}[\tilde{\sigma}\boldsymbol{\Xi} + \mathbf{O}\mathbf{O}^{\mathrm{T}}]^{-1}\mathbf{O}][\mathbf{O}^{T}\mathbf{E}]$, where $\tilde{\sigma} \geq 0$ is the augmented Lagrangian parameter, and $\boldsymbol{\Xi}$ is the identity matrix). A total of 1000 videos from four popular databases—"SumMe," "Need for Speed," and "Sports Videos in the Wild"—were used for this analysis. As shown in Supplementary Fig. 3e, the results reveal that SCARF's sensing matrix generates more accurate initializations than CUP.

Supplementary Note 5: Comparison of SCARF with sequentially time all-optical mapping photography (STAMP)

The comparison of SCARF with sequentially time all-optical mapping photography (STAMP) [24] is summarized in Supplementary Table 3. Akin to other time-stretching approaches [25-29], both SCARF and STAMP leverage time-spectrum mapping to attach temporal information in a dynamic scene to different wavelengths. The data acquisition in both modalities is built upon optical signal processing in the spectral domain, rather than in the temporal domain. As a result, both SCARF and STAMP can record ultrafast events without using ultrafast detectors. Meanwhile, the data acquisition and image reconstruction stay the same for different imaging speeds. Thus, the imaging speed can be tuned in a wide range by simply changing the duration of the linearly chirped pulse.

SCARF separates from STAMP in the following aspects. Conceptually, SCARF integrates compressed sensing with time stretching in data acquisition. This computational imaging approach results in a different system design. In particular, SCARF allows the use of a continuous chirped pulse, rather than a limited number of daughter pulses used in STAMP. From the standpoint of instrumentation, SCARF needs neither the temporal mapping device nor the spatial mapping device (SMD) in the STAMP system. The system configuration becomes much simpler. The symmetrical configuration in SCARF is also more resilient to chromatic aberration induced by the

probe pulse's bandwidth. Moreover, STAMP must record each daughter pulse at a different position on the sensor. This requirement induces an inherent trade-off issue between the frame size and sequence depth. In contrast, the intermix of spatial and temporal information in SCARF's data acquisition nullifies the need for sensor-plane division performed in STAMP, which has increased the frame size. Finally, the sequence depth of STAMP is limited by the number of channels in the periscope array of the SMD. Therefore, the design complexity and cost will be increased according to the sequence depth. In contrast, SCARF's data acquisition is independent of the sequence depth. The different system design and instrument implementation in SCARF leads to the considerate improvement in system specifications. Compared to STAMP, SCARF enhances the imaging speed by >35× and the sequence depth by 22×.

Supplementary Note 6: Details on SCARF of single ultrashort pulses transmitting through transparencies

Second harmonic generation frequency-resolved optical gating (SHG-FROG) measurements of chirped pulses

The second harmonic generation (SHG) frequency-resolved optical gating (FROG) technique [30] was implemented to characterize the linear chirp of the ultrashort laser pulses. In particular, a replica of the incident pulse was generated by a beam splitter, and then both pulses were focused on a beta barium borate crystal to generate a second harmonic signal that was only present when both pulses overlapped in space and time. An optical delay line generated variable delays between the two pulses. The resulting time-dependent second harmonic signal was recorded by a spectrometer. The pulse duration was retrieved from the measured spectrogram by a retrieval algorithm [31]. Supplementary Figs. 4a–c show the retrieved SHG-FROG measurements of the three chirped ultrashort pulses used for SCARF's proof-of-concept experiments. The reconstructed intensity and phase of the three ultrafast pulses are shown in Supplementary Figs. 4d–f, whose pulse durations are quantified to be 362 fs, 561 fs, and 739 fs, respectively.

Additional data

We carried out two additional experiments. First, a single chirped pulse [with a full-width-at-half-maximum (FWHM) duration of 561 fs] transmitting through the pattern of an "INRS" logo (Supplementary Fig. 5a). SCARF was operating at 74.9 Tfps. The full reconstructed sequence is

shown in Supplementary Movie 3. We analyzed the average intensity of each frame. Shown in Supplementary Figs. 5b, the single-shot result well agrees with the scanned SHG-FROG measurements. Finally, the linear relationship between the spectrum and time of this pulse was verified, as shown in Supplementary Figs. 5c. In the second experiment, a single chirped pulse with an FWHM duration of 739 fs was used with a maple leaf transparency (Supplementary Figs. 5d). SCARF was operating at 56.8 Tfps. The results, which are shown in Supplementary Movie 4 as well as Supplementary Figs. 5e–f, prove SCARF's real-time ultrafast imaging ability.

Supplementary Note 7: Details on SCARF of ultrafast absorption in a semiconductor

Experimental setup

Supplementary Fig. 6a shows the detailed schematic of the experimental setup for imaging femtosecond laser-induced ultrafast absorption of zinc selenide (ZnSe) by SCARF. The pump pulse had a 40-fs FWHM duration. Then, it went through a beam de-expansion stage [consisting of a 300 mm-focal-length plano-convex lens (LA1484-B, Thorlabs) and a 100 mm-focal-length plano-concave lens (LC1120-B, Thorlabs)] and an optical delay line (providing a tunable delay from -0.845 ps to 0.845 ps). Afterward, the pump pulse passed through a beam shaping stage to generate a particular pattern on a ZnSe plate with the incident angle of θ. The pump pulse induced ultrafast absorption dynamics in ZnSe, which caused a drop in its optical transmissivity [32].

The transmitted component went through the two bandpass filters (see details in Methods) for bandwidth adjustment and a variable attenuator (NDC-50C-2M, Thorlabs) for intensity adjustment. Then, this generated probe pulse illuminated the ZnSe plate to record this transient event for SCARF. Supplementary Fig. 6b shows the layout for observing this phenomenon in a single shot. The oblique incidence of the shaped pump pulse induces a delay in the onset of the absorption in 2D. In this way, the transmitted probe pulse reveals the propagation of the structured absorption front on the surface of the ZnSe plate.

Modeling the propagation of the absorption front of the ellipse on the ZnSe plate

By traversing through the beam shaping stage, the pump pulse generates a circle whose radius is denoted by r_a. Because of the oblique incidence, the generated circle is projected to an ellipse (inset in Fig. 3d). The short axis, whose direction is perpendicular to the plane of the incident, has a radius of $r_y = r_\mathrm{a}$. The long axis, within the plane of the incident, has a radius of $r_x = r_\mathrm{a}/\cos\theta$. This ellipse is expressed by

$$\frac{x^2}{r_x^2} + \frac{y^2}{r_y^2} = 1. \tag{S14}$$

Because of the flat front of the pump pulse, on the x axis, the location of the absorption front moves as a function of time by

$$x = \frac{r_{\mathrm{a}}}{\cos\theta} - \frac{ct'}{\sin\theta}. \tag{S15}$$

Here, the local time t' ranges from 0 to $(2r_{\mathrm{a}}\tan\theta/c)$. Plug Eq. (S15) into Eq. (S14), the location of the absorption front on the y axis is determined by

$$y = \sqrt{\frac{ct'}{\tan\theta}\left(2r_{\mathrm{a}} - \frac{ct'}{\tan\theta}\right)}. \tag{S16}$$

Velocities of the top and the bottom traces of the absorption front can be calculated by

$$\begin{aligned} &\mathbf{v}_{\mathrm{t}x} = \mathbf{v}_{\mathrm{b}x} = -\frac{c}{\sin\theta}\hat{\mathbf{x}}, \text{and} \\ &\mathbf{v}_{\mathrm{t}y} \text{ and } \mathbf{v}_{\mathrm{b}y} = \pm\sqrt{\frac{c}{t'\tan\theta\left(2r_{\mathrm{a}} - \frac{ct'}{\tan\theta}\right)}}\left(r_{\mathrm{a}} - \frac{ct'}{\tan\theta}\right)\hat{\mathbf{y}}. \end{aligned} \tag{S17}$$

Supplementary Note 8: Details on SCARF of ultrafast demagnetization of an alloy film

Experimental setup

Supplementary Fig. 7a shows the detailed schematic of the experimental setup of ultrafast laser-induced demagnetization of a GdFeCo alloy film. The pump pulse (40-fs pulse duration and 6.4-μJ pulse energy) was loosely focused by a 600 mm-focal-length lens to a spot with a diameter of 115 μm that was obliquely incident onto the GdFeCo sample at the angle of incidence of $\theta = 37^{\circ}$. The probe pulse (p-polarization and 0.4-μJ pulse energy) went through a mask (see the inset in Fig. 4a), which was imaged onto the sample by a tube lens and a 20× objective lens.

The reflected probe pulse was collected by the same objective lens and a tube lens to form an image at an intermediate image plane that interfaced with the polarization-resolved SCARF. As shown in the inset of Supplementary Fig. 7a, a half-wave plate (WPH10M-780, Thorlabs) and a Rochon prism (PLM 10, B.Halle Nachfl. GmbH) were placed between lens L4 and the CCD camera. Thus, the s-polarization and the p-polarization components of the reflected

probe pulse landed at two different spatial positions of the CCD camera. The polarization axis of this prism was aligned to +45° to obtain equal intensities of the two components.

A permanent magnet was placed behind the GdFeCo film. The pump pulse induced an ultrafast demagnetization process to the film, which changed the polarization angle of the reflected probe pulse. This magneto-optic Kerr effect was reflected in intensity changes in the s- and p-polarized components.

Multi-shot scanning-based experiment

To verify SCARF's result using a gold standard, we performed a scanning-based multi-shot experiment (Supplementary Fig. 7b). The sample preparation, the reflection-mode setup, and the polarization-resolved detection were kept the same as those in the SCARF's measurement. Different from the SCARF's experimental setup, the 40-fs pump pulse was focused to a spot of ~100 µm in diameter on the sample with an incident angle of ~5º, and a 40-fs unchirped pulse was used as the probe pulse. The difference between the s- and the p-polarized components of the reflected probe light was directly detected by a balanced photodiode detector (PDB220A2, Thorlabs). The step of delay time between the pump and probe pulse was set to 50 fs. Each measurement was averaged three times.

Fitting the time courses of normalized intensity difference during demagnetization

The measurements from the SCARF system and the multi-shot experiment were fitted by [33]

$$\Delta\mathrm{M}(t) = U - \mathrm{H}(t - t_{\mathrm{ON}}) \times B\left[1 - \exp\left(-\frac{t - t_{\mathrm{ON}}}{\tau_{\mathrm{dm}}}\right)\right]. \quad \text{(S18)}$$

Here, U describes the baseline. $\mathrm{H}(\cdot)$ represents the Heaviside function. t_{ON} is the onset time point of the demagnetization. The demagnetization time, τ_{dm}, is defined as the time between t_{ON} and when the normalized intensity difference reaches 63% of the maximum value. B is the amplitude constant of the function $\left[1 - \exp\left(-\frac{t - t_{\mathrm{ON}}}{\tau_{\mathrm{dm}}}\right)\right]$. Based on the fitting results, for SCARF measurements shown in Fig. 4c, the demagnetization times of Bar 1 and Bar 2 were quantified to be 187.5±18.6 fs and 186.9±19.8 fs, respectively. The onset times t_{ON} for Bar 1 and Bar 2 were determined to be -3.2±14.8 fs and 33.4±10.7 fs, respectively. Thus, the onset difference between these two areas was 36.6 fs. For the results of the multi-shot experiment, the demagnetization time was quantified to be 188.0 fs. This excellent agreement verifies the SCARF's imaging ability. This fitting was also applied to the data shown in Supplementary Fig. 7d.

Additional data

The single-shot ultrafast imaging experiment of demagnetization of an alloy film was repeated. As an example, another dataset is shown in Supplementary Fig. 7d. These data confirm the ultrafast imaging capability as well as the findings and results obtained by SCARF. They also show the stability of the demagnetization time for the used GdFeCo film. Finally, the data reveal subtle differences in the demagnetization process, showcasing the advantage of single-shot real-time femtophotography.

Supplementary Figures

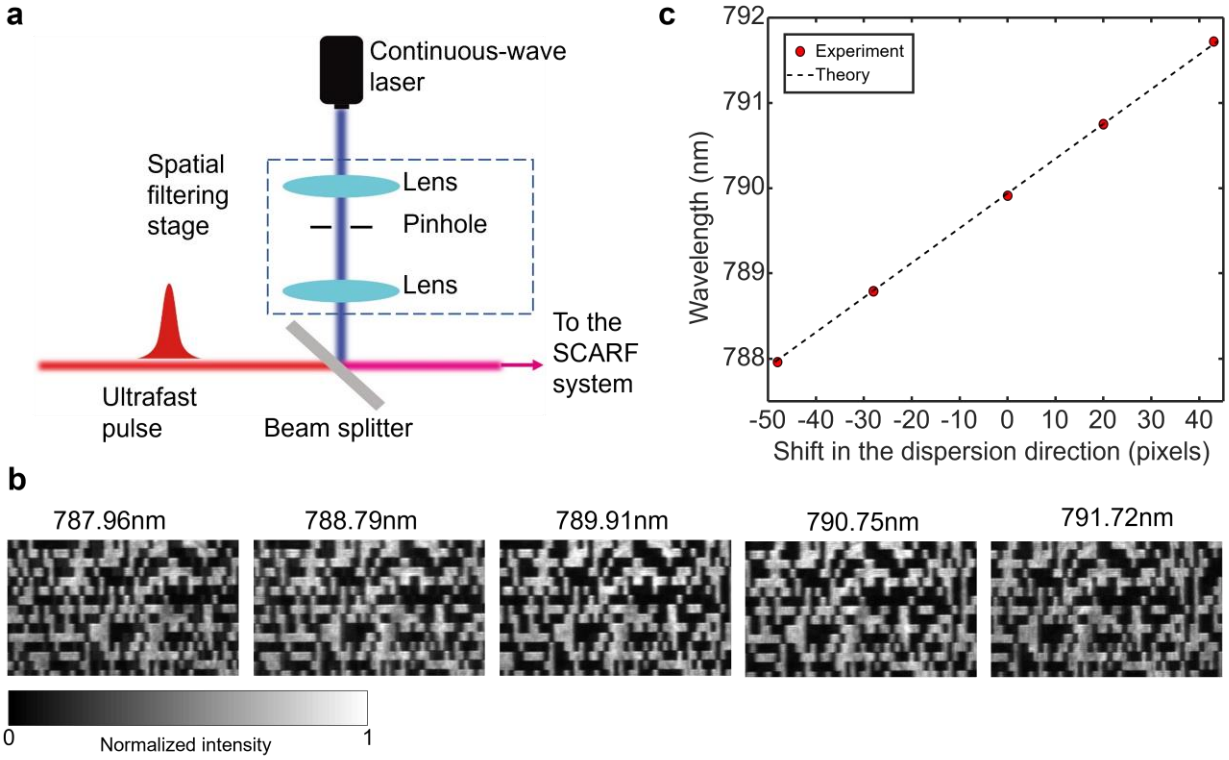

Supplementary Figure 1. Calibrating the spatial encoding operation of swept coded aperture real-time femtophotography (SCARF). (a) Schematic of the experiment setup. **(b)** Images of the encoding mask by the SCARF system illuminated by five selected wavelengths for calibration. **(c)** Shifts in the spectral shearing direction of the images acquired in (b) versus the calibration wavelengths.

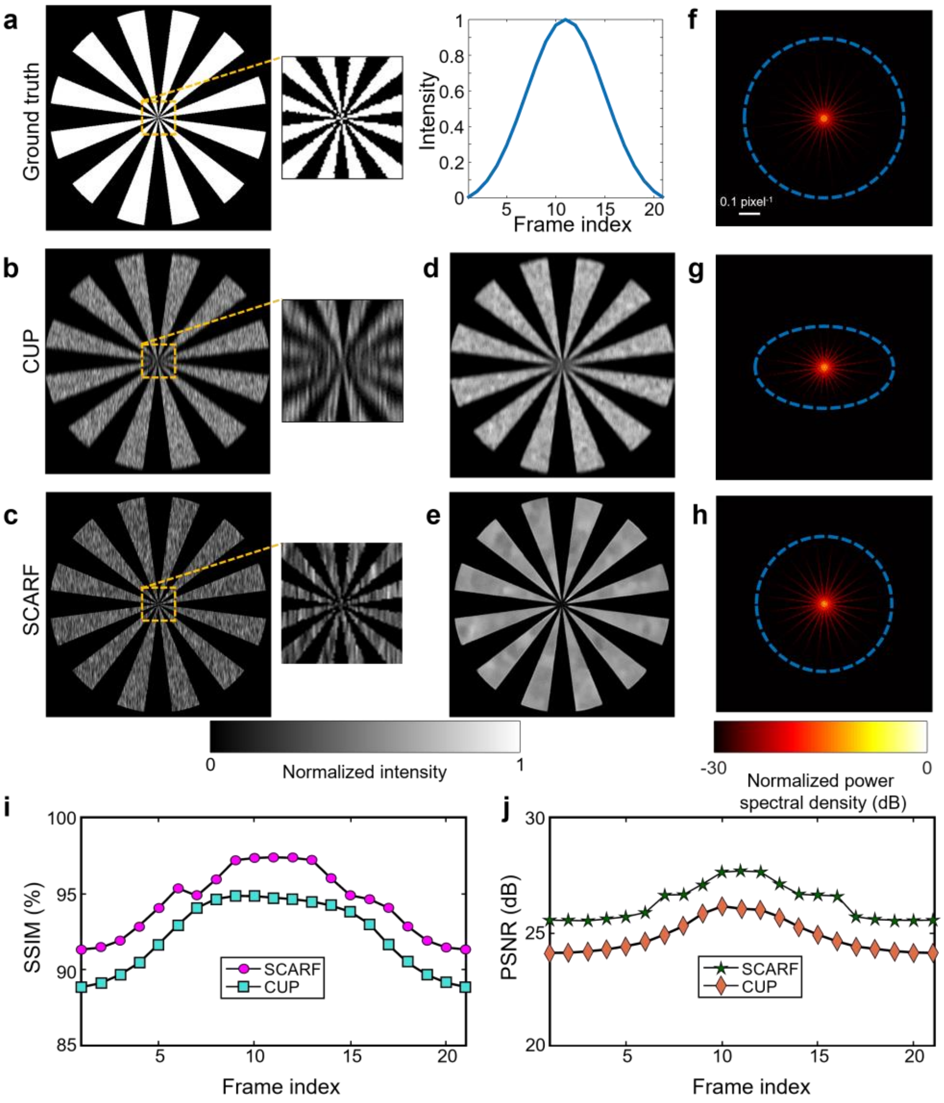

Supplementary Figure 2. Comparison of reconstructed image quality of compressed ultrafast photography (CUP) and SCARF using a static object with varying intensity. (a) Static spoke pattern (left panel) and the varying intensity (right panel) in the ground truth. Inset: Zoom-in view of the pattern center. **(b)** Snapshot generated by CUP. **(c)** Snapshot generated by SCARF. Insets in (b) and (c): Zoom-in views of the centers of the snapshots. **(d)** Representative reconstructed frame of CUP. **(e)** Representative reconstructed frame of SCARF. **(f)**–**(h)** Spatial frequency distributions of the images in the ground truth [left panel of (a)], CUP reconstruction (d), and SCARF reconstruction (e), respectively. The noise-limited bandwidth is delineated by the cyan dashed circle. **(i)**–**(j)** Structural similarity (SSIM) index and peak signal-to-noise ratio (PSNR) of each frame reconstructed by CUP and SCARF.

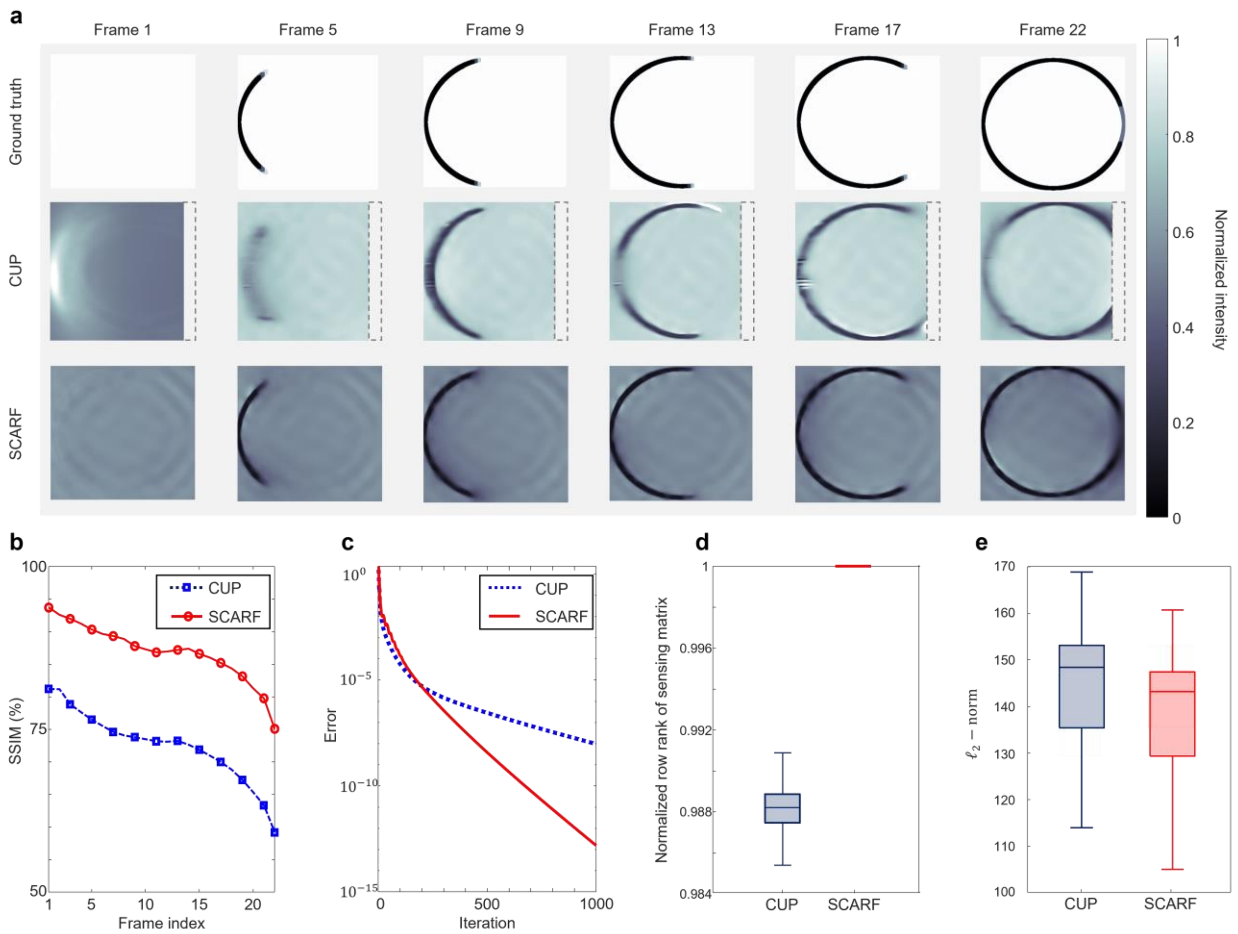

Supplementary Figure 3. Comparison of reconstructed image quality of CUP and SCARF using a moving target. (a) Six selected frames of the ground truth (first row) and the reconstructions obtained by CUP (second row) and SCARF (third row). **(b)** Comparison of structural similarity index measure (SSIM) of each reconstructed frame. **(c)** Iteration course convergence analysis using the residual norm. **(d)** Row-rank analysis for the sensing matrices. **(e)** Reconstruction error obtained by the ADMM's closed-form solution in the first iteration. In the box plots in (d) and (e), the center line represents the median, the top and bottom of the boxes represent the 75th and 25th percentiles, respectively, and the whiskers indicate the minimum and maximum values.

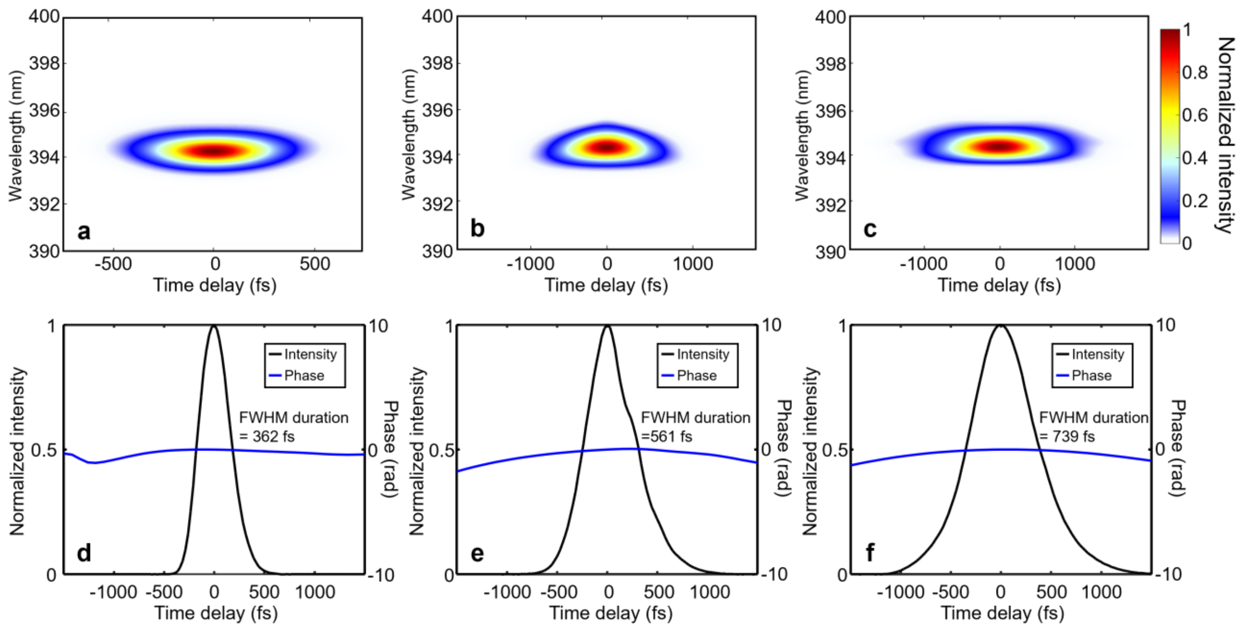

Supplementary Figure 4. Second harmonic generation frequency-resolved optical gating (SHG-FROG) measurements of the three ultrashort pulses used in SCARF's proof-of-concept experiments. (a)–(c) Retrieved SHG-FROG traces of the ultrashort pulses. **(d)–(f)** Pulse intensity and phase profiles in the time domain. FWHM, Full width at half maximum.

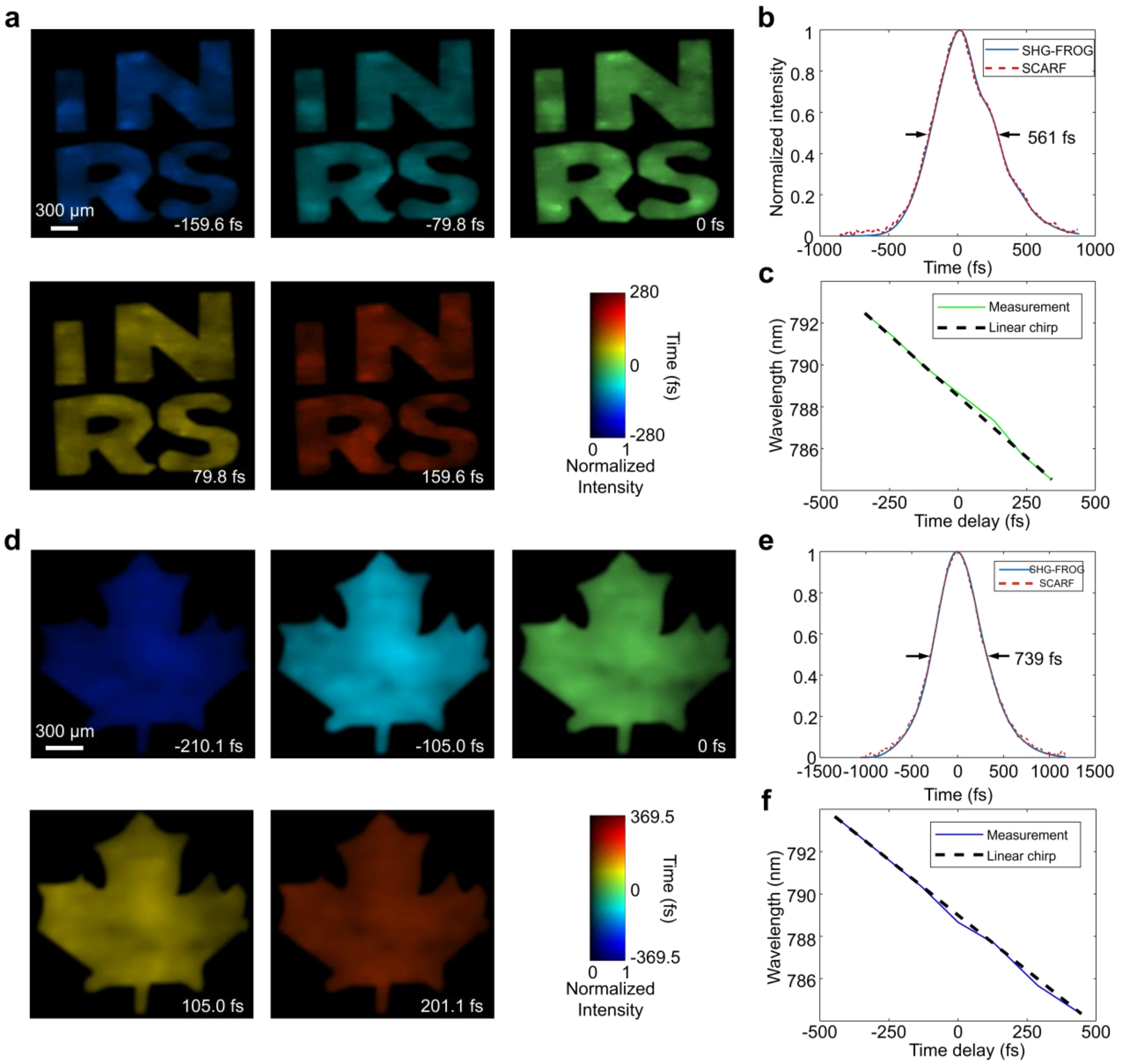

Supplemental Figure 5. Additional data of SCARF of single ultrashort pulses transmitting through transparencies. (a) Representative frames from the reconstructed movie showing a 561-fs chirped pulse transmitting through an "INRS" logo transparency imaged at 116.3 Tfps. **(b)** Time courses of the normalized average intensities for the reconstructed movie presented in (a). SHG-FROG, second harmonic generation frequency-resolved optical gating. **(c)** Relation between the wavelength and time of the chirped pulses. Black dashed lines represent perfectly linear chirping. **(d)–(f)**, As (a)–(c), but showing a 739-fs chirped pulse transmitting through a maple-leaf transparency.

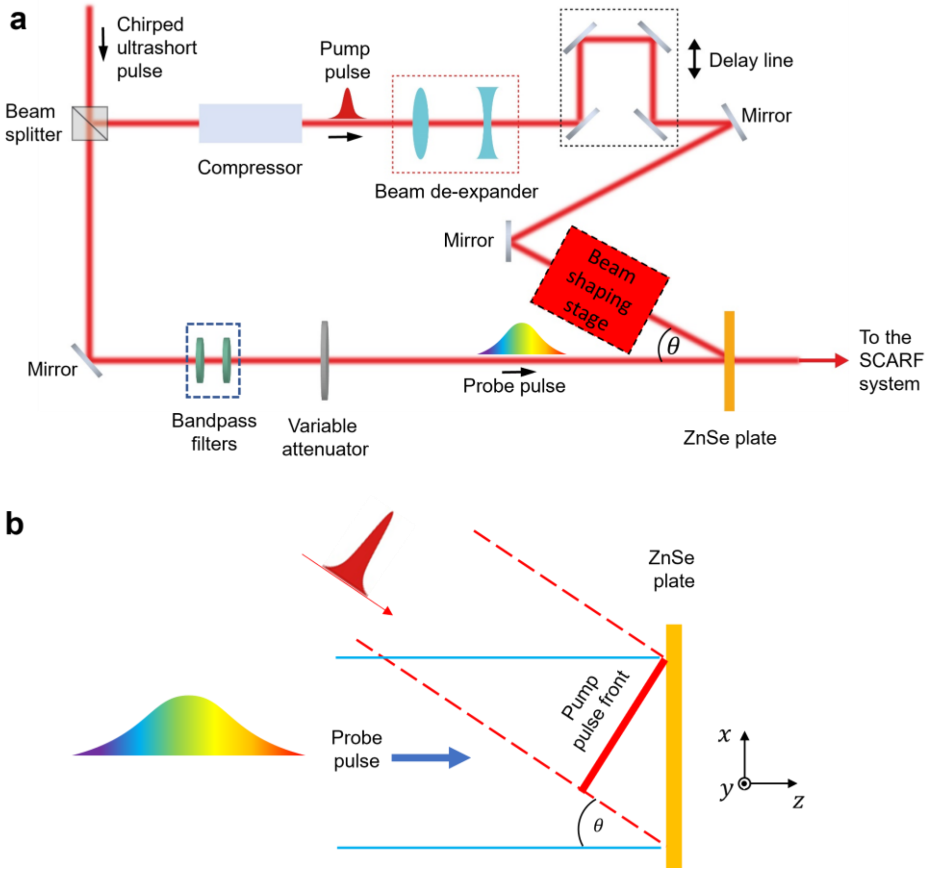

Supplementary Figure 6. Schematic (a) and geometry (b) of generating and probing the femtosecond laser-induced ultrafast absorption phenomena on a zinc selenide (ZnSe) plate.

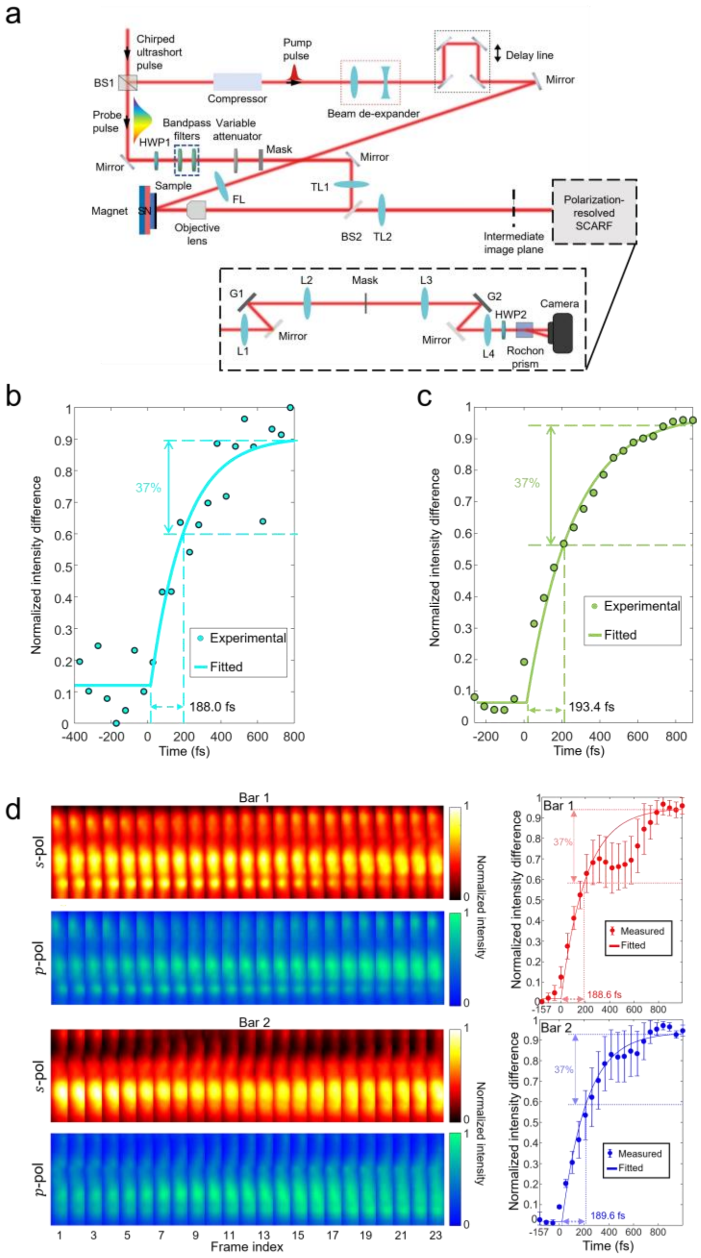

Supplementary Figure 7. Details on SCARF of ultrafast demagnetization of a GdFeCo alloy film. (a) Schematic of the experimental setup. Inset: Schematic of the polarization-resolved SCARF system. BS1–BS2, Beam splitter; G1–G2, Grating; HWP1–HWP2, Half-wave plate; FL, Focusing lens; L1–L4, Lens; P0–P2, Polarizer; TL1–TL2, Tube lens. **(b)** Time-resolved intensity difference between the s- and p-polarization light measured by the multi-shot experiment. **(c)** As (b), but showing the averaged intensity from the two selected areas in the SCARF measurement. **(d)** Another dataset of single-shot ultrafast demagnetization imaging by SCARF. The marker center and the error bar in (d) represent the mean value and the standard deviation, respectively.

Supplementary Tables

Experiment \ System parameter		Duration of the probe pulse (fs)	Imaging speed (Tfps)	Sequence depth (frames)	Spatial resolution (μm)	Field of view (mm^2)
Single ultrashort pulses transmitting through transparencies	Bar pattern	362	116.3	132	38.5	3.4×3.4
	INRS logo	561	74.9	132	46.7	2.8×2.8
	Maple leaf	739	56.8	132	41.2	3.2×3.2
Transient absorption in a semiconductor		6500	6.5	42	35.7	3.6×3.6
		530	156.3	11	33.0	0.78×0.61
Ultrafast demagnetization of an alloy film		1200	19.1	23	2.2	0.15×0.15

Supplementary Table 1. SCARF's system parameters for each experiment.

Image modality	CUST [Supp. Ref. 19]	CUSP [Supp. Ref. 20]	CACTI [Supp. Ref. 13]	MA-CS CMOS [Supp. Ref. 18]	SCARF [This work]
Maximum imaging speed	3.85 Tfps	70 Tfps	~5 kfps	200 Mfps	156.3 Tfps
Approach to recording temporal information	Mask and scene shearing	Mask and scene shearing	Mask shearing	Flutter shutter	Mask shearing
Approach to implementing coded apertures	Single static mask	Single static mask	Single moving mask or spatial light modulator	High-bandwidth circuit	Single static mask
All-optical data acquisition	Yes	No	Yes	Yes	Yes
Compression ratio	$\frac{N_t}{1+\frac{N_t}{N_y}}$	$\frac{N_t}{1+\frac{N_t}{N_y}}$	N_t	2.1	N_t
Maximum sequence depth (N_t frames)	60	140 [a]<br>980 [b]	148	32	132 [c]<br>42 [d]
Frame size ($N_x \times N_y$ pixels)	340×375	532×512	248×256	64×108	1024×1024 [e]
Data acquisition capability (Frames・Tfps)	231	9800 [a]<br>68600 [b]	0.74×10^{-6}	6.4×10^{-3}	15351.6 [f]<br>1719.3 [g]

Notes: [a] For one chirped sub-pulse as active illumination

[b] For a pulse train containing seven chirped sub-pulses as active illumination

[c] For imaging ultrafast dynamics of a static target at 116.3 Tfps

[d] For imaging an ultrafast moving target at 6.5 Tfps

[e] Maximum frame size in theory; The experimentally demonstrated frame size in this work varies from 53×67 pixels to 341×341 pixels

[f] For imaging ultrafast dynamics of a static target at 116.3 Tfps with a sequence depth of 132 frames

[g] For imaging an ultrafast moving target at 156.3 Tfps with a sequence depth of 11 frames

Supplementary Table 2. Comparison of SCARF to representative single-shot compressed temporal imaging. CACTI, coded aperture compressive temporal imaging; CUSP, compressed ultrafast spectral photography; CUST imaging, compressed ultrafast spectra-temporal imaging; MA-CS CMOS, multiple-aperture compressed-sensing CMOS. N_x and N_y, numbers of pixels in the x and y directions; N_t, sequence depth.

Image modality	STAMP<br>[Supp. Ref. 24]	SCARF<br>[This work]
Schematic	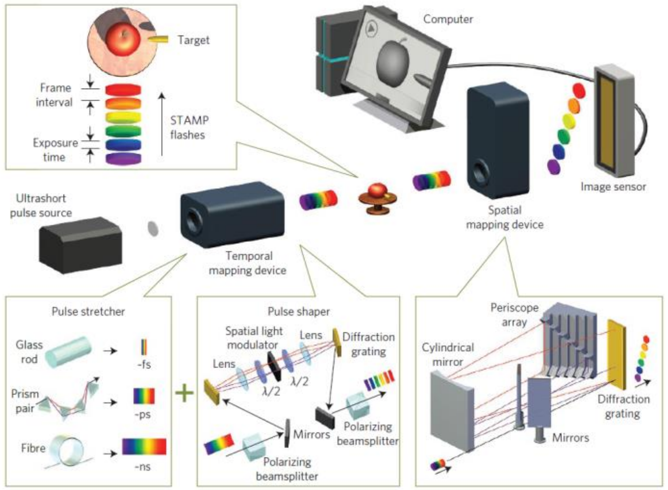	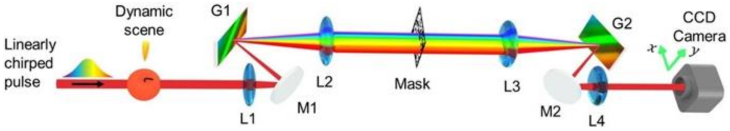<br>CCD, Charge-coupled device; G1–G2, Grating; L1–L4, Lens; M1–M2, Mirror.
Operating principle	Direct imaging	Computational reconstruction
Imaging speed	65.4 Gfps − 4.4 Tfps	6.5 Tfps − 156.3 Tfps
Sequence depth (frames)	6	11 − 132
Frame size ($N_x \times N_y$)	450 × 450	1024 × 1024[a]
Applications	(1) Laser plasma<br>(2) Phonon propagation	(1) Ultrafast absorption<br>(2) Ultrafast demagnetization

[a] Maximum frame size in theory; The maximum frame size experimentally demonstrated in this work is 341×341 pixels

Supplementary Table 3. Comparison of SCARF to sequentially timed all-optical mapping photography (STAMP).

Supplementary References

1 Diels, J.-C. & Rudolph, W. *Ultrashort laser pulse phenomena*. (Elsevier, 2006).

2 Candes, E. J. & Wakin, M. B. An Introduction To Compressive Sampling. *IEEE Signal Processing Magazine* **25**, 21-30 (2008).

3 Zhu, L. *et al.* Space- and intensity-constrained reconstruction for compressed ultrafast photography. *Optica* **3**, 694-697 (2016).

4 Otsu, N. A threshold selection method from gray-level histograms. *IEEE Transactions on Systems, Man, and Cybernetics* **9**, 62-66 (1979).

5 Boyd, S., Parikh, N. & Chu, E. *Distributed optimization and statistical learning via the alternating direction method of multipliers*. (Now Publishers Inc, 2011).

6 Burger, H. C., Schuler, C. J. & Harmeling, S. Image denoising: Can plain neural networks compete with BM3D? *2012 IEEE Conference on Computer Vision and Pattern Recognition*, 2392-2399 (2012).

7 Danielyan, A., Katkovnik, V. & Egiazarian, K. BM3D frames and variational image deblurring. *IEEE Transactions on Image Processing* **21**, 1715-1728 (2011).

8 Dabov, K., Foi, A., Katkovnik, V. & Egiazarian, K. Image denoising by sparse 3-D transform-domain collaborative filtering. *IEEE Transactions on Image Processing* **16**, 2080-2095 (2007).

9 Lai, Y. *et al.* Single ‐ Shot Ultraviolet Compressed Ultrafast Photography. *Laser & Photonics Reviews* **14**, 2000122 (2020).

10 Guizar-Sicairos, M., Thurman, S. T. & Fienup, J. R. Efficient subpixel image registration algorithms. *Optics Letters* **33**, 156-158 (2008).

11 Reddy, D., Veeraraghavan, A. & Chellappa, R. in *CVPR 2011*. 329-336 (IEEE).

12 Liu, D. *et al.* Efficient space-time sampling with pixel-wise coded exposure for high-speed imaging. *IEEE Transactions on Pattern Analysis and Machine Intelligence* **36**, 248-260 (2013).

13 Llull, P. *et al.* Coded aperture compressive temporal imaging. *Optics Express* **21**, 10526-10545 (2013).

14 Koller, R. *et al.* High spatio-temporal resolution video with compressed sensing. *Optics Express* **23**, 15992-16007 (2015).

15 Hu, C., Huang, H., Chen, M., Yang, S. & Chen, H. Video object detection from one single image through opto-electronic neural network. *APL Photonics* **6**, 046104 (2021).

16 Qiao, M., Liu, X. & Yuan, X. Snapshot temporal compressive microscopy using an iterative algorithm with untrained neural networks. *Optics Letters* **46**, 1888-1891 (2021).

17 Yuan, X., Liu, Y., Suo, J. & Dai, Q. in *Proceedings of the IEEE/CVF Conference on Computer Vision and Pattern Recognition*. 1447-1457.

18 Mochizuki, F. *et al.* Single-event transient imaging with an ultra-high-speed temporally compressive multi-aperture CMOS image sensor. *Optics Express* **24**, 4155-4176 (2016).

19 Lu, Y., Wong, T. T. W., Chen, F. & Wang, L. Compressed Ultrafast Spectral-Temporal Photography. *Physical Review Letters* **122**, 193904 (2019).

20 Wang, P., Liang, J. & Wang, L. V. Single-shot ultrafast imaging attaining 70 trillion frames per second. *Nature Communications* **11**, 2091 (2020).

21 Qi, D. *et al.* Single-shot compressed ultrafast photography: a review. *Advanced Photonics* **2**, 014003 (2020).

22 Gao, L., Liang, J., Li, C. & Wang, L. V. Single-shot compressed ultrafast photography at one hundred billion frames per second. *Nature* **516**, 74-77 (2014).

23 Chan, S. H., Wang, X. & Elgendy, O. A. Plug-and-play ADMM for image restoration: Fixed-point convergence and applications. *IEEE Transactions on Computational Imaging* **3**, 84-98 (2016).

24 Nakagawa, K. *et al.* Sequentially timed all-optical mapping photography (STAMP). *Nature Photonics* **8**, 695-700 (2014).

25 Goda, K., Tsia, K. & Jalali, B. Serial time-encoded amplified imaging for real-time observation of fast dynamic phenomena. *Nature* **458**, 1145-1149 (2009).

26 Lei, C., Guo, B., Cheng, Z. & Goda, K. Optical time-stretch imaging: Principles and applications. *Applied Physics Reviews* **3**, 011102 (2016).

27 Wu, J.-L. *et al.* Ultrafast laser-scanning time-stretch imaging at visible wavelengths. *Light: Science & Applications* **6**, e16196-e16196 (2017).

28 Zeng, X. *et al.* High-spatial-resolution ultrafast framing imaging at 15 trillion frames per second by optical parametric amplification. *Advanced Photonics* **2**, 056002 (2020).

29 Suzuki, T. *et al.* Single-shot 25-frame burst imaging of ultrafast phase transition of Ge2Sb2Te5 with a sub-picosecond resolution. *Applied Physics Express* **10**, 092502 (2017).

30 Kane, D. J. & Trebino, R. Characterization of arbitrary femtosecond pulses using frequency-resolved optical gating. *IEEE Journal of Quantum Electronics* **29**, 571-579 (1993).

31 Trebino, R. & Kane, D. J. Using phase retrieval to measure the intensity and phase of ultrashort pulses: frequency-resolved optical gating. *JOSA A* **10**, 1101-1111 (1993).

32 Leblanc, A. *et al.* Phase-matching-free pulse retrieval based on transient absorption in solids. *Optics Express* **27**, 28998-29015 (2019).

33 Hennecke, M. *et al.* Angular momentum flow during ultrafast demagnetization of a ferrimagnet. *Physical Review Letters* **122**, 157202 (2019).
