# Supplementary material for: Swept coded aperture real-time femtophotography
Source: Nat Commun. 2024 Feb 21;15:1589. doi: 10.1038/s41467-024-45820-z (PMC10882056; doi:10.1038/s41467-024-45820-z)
Supplement: Supplementary file 3 — Description of Additional Supplementary Files [file 41467_2024_45820_MOESM3_ESM.docx]

**Description of Additional Supplementary Files**

**Supplementary Movie 1:** Animation of the operation of the SCARF system.

**Supplementary Movie 2:** SCARF of a single ultrafast pulse transmitting through transparency with a bar pattern at 116.3 Tfps.

**Supplementary Movie 3:** SCARF of a single ultrafast pulse transmitting through transparency with an “INRS” logo pattern at 74.9 Tfps.

**Supplementary Movie 4:** SCARF of a single ultrafast pulse transmitting through transparency with a maple leaf pattern at 56.8 Tfps.

**Supplementary Movie 5:** SCARF of ultrafast absorption dynamics of an elliptical pattern on a ZnSe plate at 6.5 Tfps.

**Supplementary Movie 6:** SCARF of ultrafast absorption dynamics of a line pattern on a ZnSe plate at 156.3 Tfps.

**Supplementary Movie 7:** SCARF of ultrafast demagnetization of a GdFeCo film at 19.1 Tfps
